# Computed tomography attenuation in differential diagnosis of transudative and exudative pleural effusions

**DOI:** 10.1016/j.clinsp.2024.100463

**Published:** 2024-08-06

**Authors:** Aziz Gümüş, Neslihan Özçelik, Bilge Yılmaz Kara, Nur Hürsoy, Neşe Merve Güner Zırıh, Songül Özyurt, Ünal Şahin

**Affiliations:** a Recep Tayyip Erdogan University, Faculty of Medicine, Department of Chest Disease, Rize, Turkey; b Recep Tayyip Erdogan University, Faculty of Medicine, Department of Radiology, Rize, Turkey

**Keywords:** Attenuation, Exudate, Hounsfield Units, Pleural effusion, Transudate

## Abstract

**Objective:**

Pleural effusion is a common medical problem. It is important to decide whether the pleural fluid is a transudate or an exudate. This study aims to measure the attenuation values of pleural effusions on thorax computed tomography and to investigate the efficacy of this measurement in the diagnostic separation of transudates and exudates.

**Materials and methods:**

380 cases who underwent thoracentesis and thorax computed tomography with pleural effusion were classified as exudates or transudates based on Light's criteria. Attenuation measurements in Hounsfield units were performed through the examination of thorax computed tomography images.

**Results:**

380 patients were enrolled (39 % women), the mean age was 69.9 ± 15.2 years. 125 (33 %) were transudates whereas 255 (67 %) were exudates. The attenuation values of exudates were significantly higher than transudates (15.1 ± 5.1 and 5.0 ± 3.4) (p < 0.001). When the attenuation cut-off was set at ≥ 10 HU, exudates were differentiated from transudates at high efficiency (sensitivity is 89.7 %, specificity is 94.4 %, PPV is 97 %, NPV is 81.9 %). When the cut-off value was accepted as < 6 HU, transudates were differentiated from exudates with 97.2 % specificity.

**Conclusion:**

The attenuation measurements of pleural fluids can be considered as an efficacious way of differentiating exudative and transudative pleural effusions.

## Highlights

• Pleural effusion is a common medical problem that occurs as a complication of many diseases.
• The first thing that needs to be done is to decide whether the pleural fluid, obtained by thoracentesis, is a transudate or an exudate.
• Attenuation measurements via thorax CT can be used to differentiate exudates from transudates.

## Introduction

Pleural effusions occur as a result of an imbalance between pleural fluid production and absorption. In clinical practice, they are frequently encountered as a complication of many diseases.^1^ Congestive Heart Failure (CHF) is reported to be the most common cause of pleural effusions. Malignant Pleural Effusions (MPE) and Parapneumonic Effusions (PPE) are other common causes.^1^ The annual incidence of pleural effusions is as high as 3‒5/1000 cases per year. A definitive diagnosis of the disease, causing pleural effusion is possible after systematic evaluation and many interventional procedures. The first question that needs to be answered for a patient with pleural effusion is whether the fluid is transudative or exudative in nature. Light criteria are often used as an efficacious tool while dealing with pleural effusions. They were defined by Light et al. in 1972 and have high sensitivity and specificity in differentiating transudative and exudative pleural effusions.^2^ If thoracentesis shows a transudative pleural fluid based on Light's criteria consistent with clinical as well as radiological findings, no further investigations are required. There are many causes of transudative effusions with CHF, renal failure, hypoalbuminemia, and atelectasis being the primary ones. If exudative fluid is detected by thoracentesis, further investigations are needed to reach the etiological diagnosis. Although thoracentesis is relatively safe, it is an invasive procedure that can cause minor complications such as pain, cough, and subcutaneous hematoma as well as certain major complications such as pneumothorax, hemothorax, and organ injuries.^3^, ^4^, ^5^ Presently there is a need to develop an efficient and non-invasive method to avoid these complications.

Chest radiography, ultrasonography, and Computed Tomography (CT) are the most commonly used imaging modalities in the diagnostic process of pleural effusions. Thorax computed tomography allows a detailed evaluation of fluid-related features such as the amount of pleural effusion, investigation of pleural nodule and thickening, presence of loculation, and accompanying conditions such as pneumonia and malignancy that can cause pleural effusion.^6^ The attenuation values of effusions in Hounsfield Units (HU) are measured on the axial sections of thorax CT. Hounsfield units are a measure of X-Ray attenuation in CT images. The HU value of water is accepted as zero under normal pressure and temperature. The contents of transudative and exudative pleural fluids are different. Therefore, it is expected that there will be a difference in attenuation values that helps to distinguish them from each other. There are a limited number of studies on this subject which were conducted on a small number of patients. In this context, this study aims to investigate the efficacy of attenuation measurements via thorax CT for differentiation of transudative and exudative pleural effusions and to reveal the factors affecting attenuation.

## Materials and methods

The patients over the age of 18, who had pleural effusions in their thorax CT and underwent thoracentesis within two days of pleural effusion diagnosis were included in the study. The data regarding the period between January 2018 and March 2022 were collected retrospectively. Initially, a differentiation was made between transudative and exudative effusions according to Light's criteria by using biochemical markers. Etiological factors were investigated through other diagnostic procedures such as cytopathological and bacteriological examinations as well as echocardiography. Both non-contrast and contrast-enhanced thorax CT images were examined. The cases with undetermined pleural fluid etiology and those with artifacts on CT images that would affect the measurement were excluded from the study.

### Attenuation measurement in Hounsfield Units (HU) using multislice CT

**Fig. 1:**
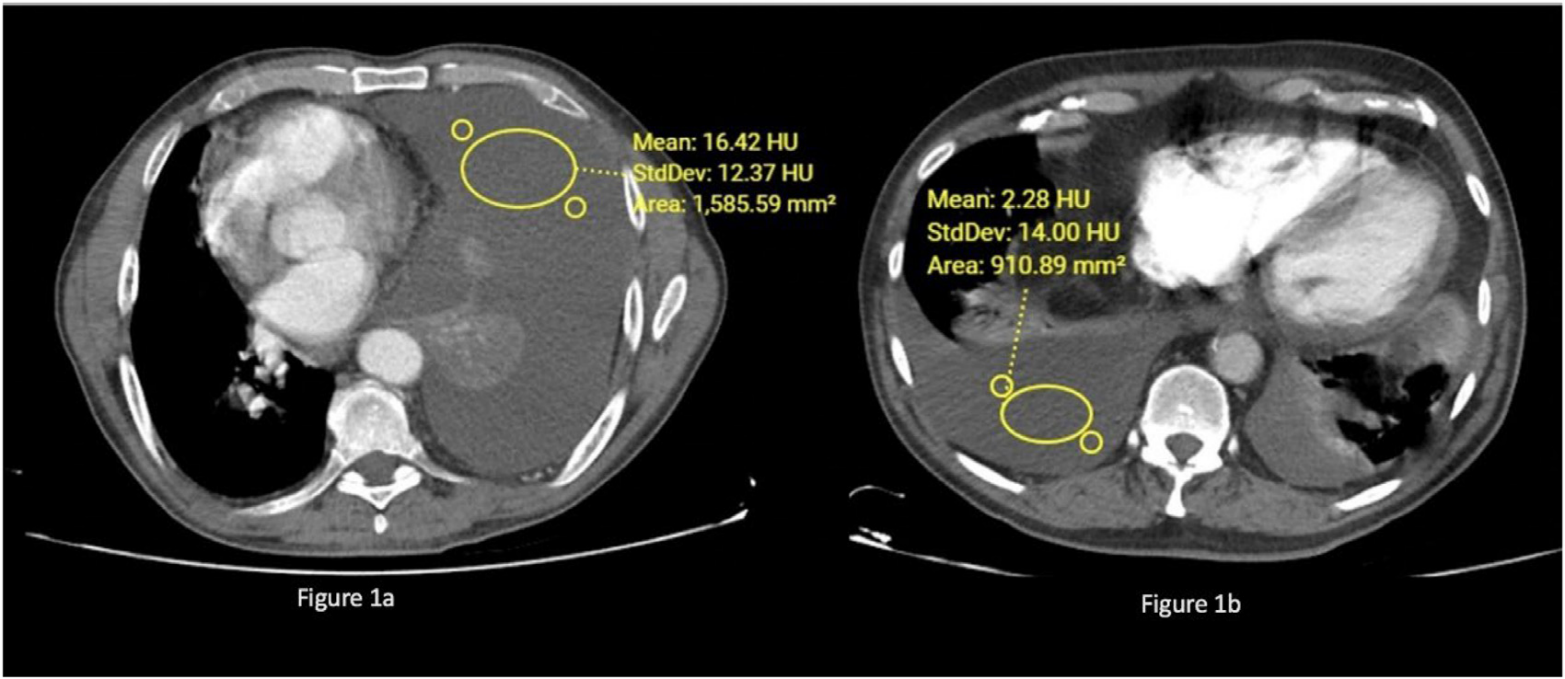
(a) The mean attenuation value of a unilateral pleural effusion due to lung adenocarcinoma in axial sections of contrast-enhanced thorax CT of a 63-year-old patient was measured as 16.42 HU. (b) The mean pleural fluid attenuation value of the bilateral pleural effusion of a 60-year-old patient with CHF was measured as 2.28 HU in axial sections of contrast-enhanced thorax CT.

The CT examinations were performed using a 128 detectors General Electric Discovery CT750 HD CT device. In CT images, the thickness of the pleural fluid was measured using the axial sections with the highest amount of pleural fluid in the posterior-anterior diameter. The measurements were made in HU using the Region Of Interest (ROI) tool. For each area selected, 3 adjacent sections were analyzed per case. The mean of the three measurements was recorded as the attenuation value of the pleural fluid (Fig. 1 a and b). Particular attention was paid not to including the lung, rib and pleural thickening areas during the measurements.

### Statistical analysis

All statistical analyses were performed using the IBM-SPSS program (SPSS version 21; SPSS Inc., Chicago, IL, USA). Continuous variables were given as mean ± standard deviations and categorical variables were given in percentages (%). The chi-Square test was used to compare categorical variables. The Student-*t* test was used to compare the two groups. The Pearson correlation analysis method was employed to analyze the relationship between the variables. Independent variables affecting the pleural HU value were determined by linear regression analysis. Receiver Operating Characteristic (ROC) analysis was performed to find the cut-off value at which the specificity and sensitivity for the exudate were optimal. Statistical significance was defined as p < 0.05.

## Results

The study included 380 patients 149 (39 %) women. The mean age of the patients was 69.9 ± 15.2 (20‒107) years. A total of 125 (33 %) pleural effusions were found to be transudates, whereas 255 (67 %) were found to be exudates. The etiologies of transudative fluids were valvular heart diseases for 61 patients, systolic heart failure for 39 patients, atelectasis for 12 patients, hypoalbuminemia for 7 patients and other causes for 6 patients when given in order of frequency. The etiologies of exudative fluids were metastatic malignancies for 158 patients, pneumonia for 60 patients, tuberculosis for 14 patients, and other causes for 23 patients. Table 1 shows the demographic, radiological and biochemical characteristics of the patients. Attenuation values of exudative fluids were found to be significantly higher than transudative fluids (15.1 ± 5.1 and 5.0 ± 3.4, respectively, p < 0.001). No significant difference was detected between the attenuation values of MPE, PPE and Tuberculous Pleurisy (TP) which are common causes of exudative fluids (14.8 ± 4.9, 15.6 ± 4.9 and 14.4 ± 3.7, respectively, p = 0.398).

**Table 1:** Demographic, radiological, and biochemical characteristics of patients with transudative and exudative pleural fluids.

	**Transudative effusion** **(n** = **125)**	**Exudative effusion** **(n** = **255)**	**p-value**
**Age (years)**	77.7 ± 11.1 (46‒97)	66.4 ± 15.5 (20‒95)	<0.001
**Gender (female/male)**	49/76	99/156	0.954
**Effusion size (mm)**	54 ± 22	65 ± 28	0.018
**Attenuation (HU)**	5.0 ± 3.4 (-5‒15.5)	15.1 ± 5.1 (2‒36)	<0.001
**Pleural protein (gr/L)**	22.2 ± 5.9	44.7 ± 9.7	<0.001
**Pleural albumin (gr/L)**	12.2 ± 3.6	25.0 ± 6.1	<0.001
**Pleural LDH**	113 ± 72	681 ± 385	<0.001
**Pleural cholesterol (mg/dL)**	30 ± 13	92 ± 32	<0.001
**Pleural triglyceride (mg/dL)**	18 ± 9	45 ± 17	0.009
**Pleural leukocyte**	542 ± 185	3544 ± 1880	0.001
**Pleural hematocrit (%)**	0.94 ± 0.14	1.29 ± 1.64	0.010
**Pleural glucose (mg/dL)**	160 ± 69	108 ± 57	<0.001
**Pleural potassium (mg/dL)**	3.6 ± 0.6	3.7 ± 0.5	0.634
**Pleural sodium (mg/dL)**	136 ± 7	135 ± 7	0.782
**Pleural calcium (mg/dL)**	4.4 ± 0.2	4.5 ± 0.6	0.700
**Pleural bilirubin (mg/dL)**	0.47 ± 0.30	1.27 ± 0.96	<0.001

mm, milimeter; HU, Hounsfield Unit; LDH, Lactate Dehydrogenase.

Data are given as mean ± standard deviation and/or minimum‒maximum.

**Fig. 2:**
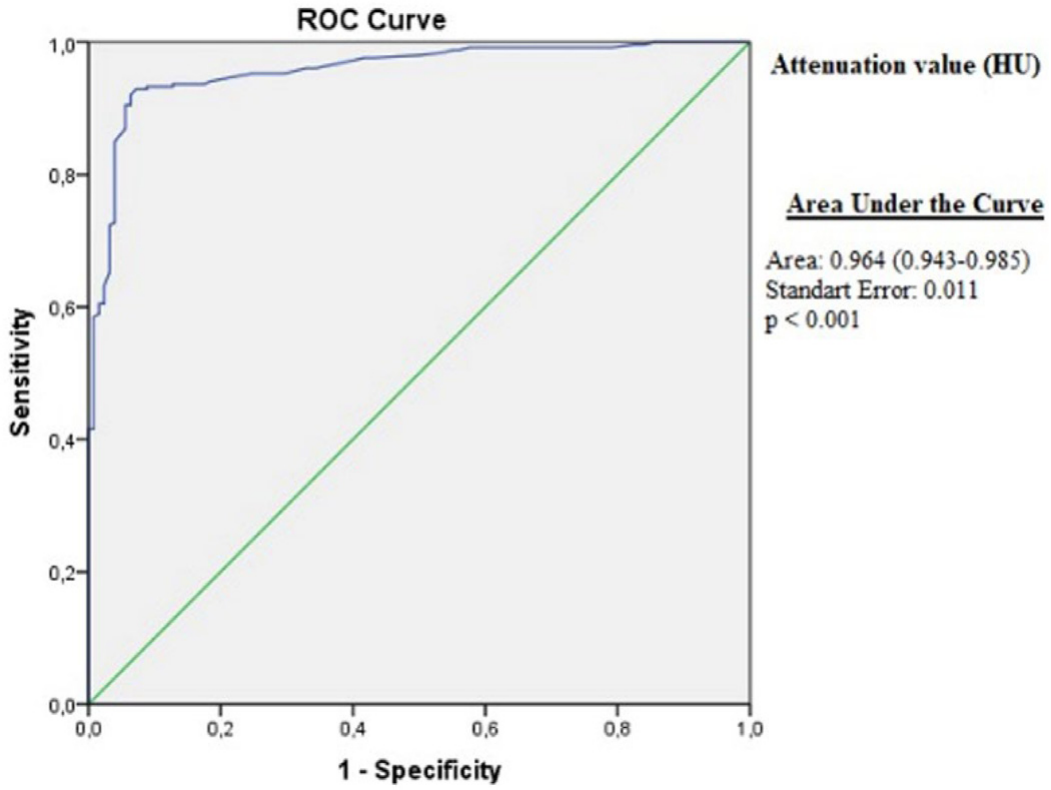
ROC analysis of attenuation values (AUC = 0.964, 95 %CI 0.943‒0.985, Standard Error = 0.011, p < 0.001).

The attenuation value had a high efficacy in differentiating between the exudative pleural effusions from transudates in the ROC analysis (Fig. 2).

Specificity, sensitivity, Positive Predictive Value (PPV) and Negative Predictive Value (NPV) were calculated at different cut-off values to reveal the efficacy of attenuation values in the differentiation of transudates and exudates (Fig. 2). When the cut-off value was accepted as ≥ 10 HU, exudative fluids were differentiated from transudative fluids with high efficacy (sensitivity: 89.7 %, specificity: 94.4 %, PPV: 97 % and NPV: 81.9 %). It was also possible to differentiate transudative fluids from exudative fluids with high efficacy when the cut-off value was accepted as < 6 HU (sensitivity: 60.8 %, specificity: 97.2 %, PPV: 91.6 % and NPV: 83.4 %**) (**Table 2**).**

**Table 2:** The efficacy of different attenuation levels of pleural fluid in differentiating between exudates and transudates.

**Cut-off value**	**Sensitivity %**	**Specificity %**	**PPD %**	**NPD %**
≥ 6	97.2	60.8	83.4	91.6
≥ 8	93.2	88.8	94.4	86.7
≥ 10	89.7	94.4	97.0	81.9
≥ 12	79.4	96	97.6	69.8

**Fig. 3:**
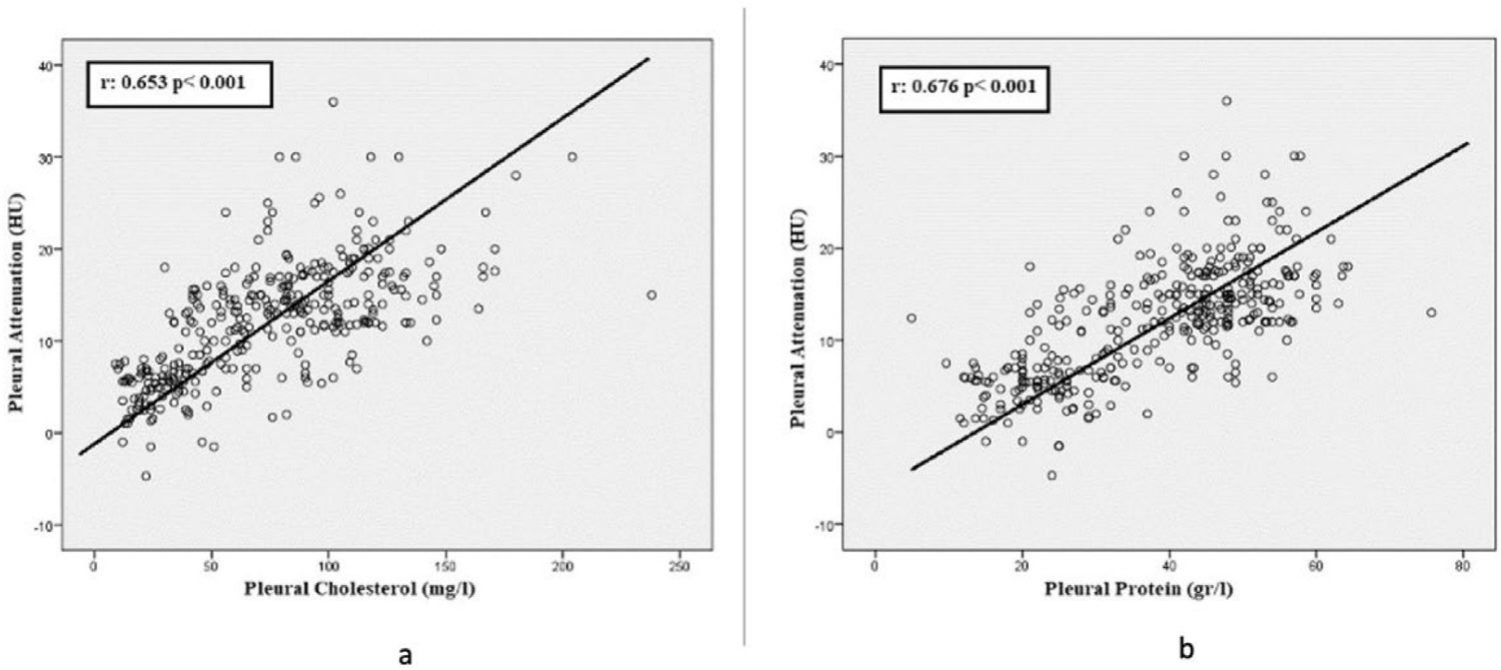
(a) The scatter plot graph showing the positive correlation between pleural attenuation values and pleural protein values. (b) The scatter plot graph showing the positive correlation between pleural attenuation values and pleural cholesterol values.

Pearson correlation analysis was performed to determine the relationship between the attenuation values of pleural fluids and other variables. A positive correlation was found between the attenuation values and pleural protein (*r* = 0.676, p < 0.001), Pleural Lactate Dehydrogenase (LDH) (*r* = 0.222, p < 0.001), pleural cholesterol (*r* = 0.653, p < 0.001), pleural hematocrit (*r* = 0.203, p = 0.001), pleural WBC (*r* = 0.139, p = 0.010), pleural bilirubin (*r* = 0.151, p = 0.046) and pleural potassium (*r* = 0.164, p = 0.033) values. A negative correlation was found between the pleural attenuation and the patient's age (*r* = -0.244, p < 0.001) and pleural glucose levels (*r* = -0.279, p < 0.001). There were no significant correlations between the attenuation values and pleural triglyceride (*r* = 0.054, p = 0.327), pleural sodium (*r* = -0.041, p = 0.760), and pleural calcium (*r* = -0.058, p = 0.674) levels. The attenuation values had the strongest correlation with pleural protein and pleural cholesterol levels (Fig. 3a and b).

Pleural cholesterol levels and pleural protein levels are detected as independent variables that affect the attenuation value in the linear regression analysis (Table 3).

**Table 3:** The variables affecting the pleural attenuation value by linear regression analyses.

	**Unstandardized coefficients**		**Standardized coefficients**	***t***	**p-value**
	**B**	**Std. Error**	**Beta**		
Constant	−6.553	4.114		−1.593	0.114
Age	0.030	0.029	0.076	1.030	0.305
Pleural glucose	0.002	0.009	0.017	0.211	0.833
**Pleural protein**	0.156	0.060	0.352	2.620	**0.010**
**Pleural cholesterol**	0.063	0.020	0.418	3.138	**0.002**
Pleural hematocrit	0.912	1.289	0.057	0.707	0.481
Pleural potassium	1.208	0.736	0.115	1.641	0.104
Pleural bilirubin	−0.395	0.437	−0.072	−0.905	0.368
Pleural leukocyte	−0.006	<0.001	0.005	0.065	0.949

## Discussion

This study showed that the attenuation values measured via CT were significantly higher in exudative fluids compared to transudative fluids. In ROC analysis, the attenuation values were highly efficacious in differentiating transudates from exudates (Area Under the Curve [AUC = 0.964], 95 % CI 0.943‒0.985, Standard error = 0.011, p < 0.001). When the cut-off value was accepted as ≥ 10 HU, the exudative effusions could be differentiated from transudative fluids with 94.4 % specificity and 97 % PPV. When the cut-off value for attenuation was accepted as < 6 HU, it was possible to differentiate transudates from exudates with 97.2 % specificity and 91.6 % PPD. In the linear regression analysis, pleural cholesterol and pleural protein levels were found to be independent predictors of attenuation levels. The attenuation measurements were similar in MPE, PPE and TP, which are the most common causes of exudative pleural effusions, and therefore, they could not be used for differentiation.

There are a limited number of studies that evaluate the characterization of pleural fluids and the differentiation between exudates and transudates based on the attenuation measurements of CT images. A retrospective study by Shaili et al., which examined 21 transudatory and 79 exudative cases with pleural effusion reported that the attenuation value of exudative fluids (31.9 ± 15.2 HU) was higher than that of transudative fluids (24.8 ± 9.9 HU, p = 0.047). In this study, the cut-off value for exudative effusions was accepted as ≥ 24.5 HU. The sensitivity and specificity of this cutoff value were 65 % and 47.6 %, respectively, which is considered as a moderate level. Hence, it was not recommended to be used for differentiating exudates from transudates.^7^ The study by Yalçın et al. included 128 patients (33 transudates and 95 exudates), and showed that the attenuation values of exudative fluids (8.82‒7.04 HU) were significantly higher than transudative fluids (2.91‒8.53 HU) (p < 0.001). In this study, when the cut-off value was accepted as > 5 HU to differentiate the exudate from the transudate by ROC analysis, sensitivity was found to be 72 %, specificity was 70 %, PPV was 87 % and NPV was 46 %.^8^ Nandalur et al. conducted a retrospective study with 145 patients. They reported that the attenuation values of exudative pleural effusions (17.1±4.4 HU) were significantly higher than transudative fluids (12.5 ± 6.3 HU, p = 0.001). They stated that the efficacy of attenuation in differentiating exudative and transudative effusions in ROC analysis was moderate (AUC = 0.775; 95 % CI 0.699‒0.851).^9^ Although the attenuation values of the exudative effusions were higher in the three studies described above, none of them suggested the use of CT for attenuation measurement in the differentiation of transudative and exudative pleural effusions. This is because of the moderate specificity and sensitivity and the overlapping attenuation values.

In a study by Ramya et al. the attenuation measurements with CT scan were performed in 80 patients (24 transudates, 56 exudates), and the mean attenuation of exudative fluids (14.65 ± 6.07) was significantly higher than that of transudative fluids (4.66 ± 2.29, p < 0.01). When the CT attenuation cut-off was accepted as < 8 HU, they measured sensitivity as 91.6 %, specificity as 82.7 %, PPV as 73.3 %, and NPV as 96 % for transudative fluids.^10^ Sharma et al. reported that pleural fluid attenuation values were significantly higher in exudates (16.5±.7 HU) compared to transudates (11.6 ± 0.57 HU, p = 0.0001).^11^ Another study including 106 patients reported that the attenuation of exudative fluids (median: 12.5 [4‒33]) was significantly higher than transudative fluids (median: 5 [2‒15], p < 0.001). When the threshold for exudative effusions was accepted as ≥8.5 HU, the sensitivity and specificity were found to be 85 % and 86.7 %, respectively.^12^ These three studies emphasized that the CT attenuation measurements can differentiate between transudates and exudates. The results of this study were in line with the results of these studies.

In a study of dogs, the median attenuation was found 19.22 (8.23‒37.66) HU in exudative fluids and 11.20 (-2.52‒16.59) HU in transudative fluids. The difference was found to be significantly higher in favor of exudative fluids (p < 0.001).^13^

Abramowitz et al. conducted a study in which they measured the attenuation of pleural fluids in CT images of 100 patients, 22 of whom were transudates and 78 were exudates. They reported the HU value of the transudates as 10.1 ± 6.9 and as 7.2 ± 9.4 (p = 0.24) for exudates. Correlation analysis revealed a weak but significant positive relationship between the attenuations and the pleural protein levels (*r* = 0.22, p = 0.003), but no relationship was found between the attenuations and the pleural LDH levels (*r* = -0.06, p = 0.52). As a result, in this study, it was concluded that the attenuation measurements via thorax CT do not have any potential value in the characterization of pleural fluids.^14^

One of the most striking and newly emphasized findings in the present study is the determination of pleural protein and pleural cholesterol levels as factors affecting the attenuation measurements. Attenuation measurement via CT is a density measurement. As the density increases, the attenuation also increases. The authors believe that, as the high molecular weight components of pleural fluid such as albumin, cholesterol, and other proteins, which are generally the most abundant ingredients increase, the attenuation also increases.

## Conclusion

As a result, attenuation measurements via thorax CT can be used to differentiate exudates from transudates. The independent factors affecting the attenuation values are pleural cholesterol and protein levels. Pleural effusions with an attenuation value of ≥10 HU should be considered exudative effusions and necessary further investigations should be performed. The fluids with an attenuation value < 6 HU are quite likely to be transudative. If the clinical and radiological features of a patient are compatible with a transudative effusion, the disease-causing the pleural effusion can be diagnosed and its possible complications can be avoided without thoracentesis. However, there is a need for comprehensive studies on this subject.

### Ethics approval

The study was approved by the local ethics committee (2021/43).

### Data availability statement

The datasets are available from the corresponding author upon reasonable request.

### Authors’ contributions

Conceptualization: Aziz Gumus, Neslihan Ozcelik.

Data curation: Aziz Gumus, Neslihan Ozcelik, Songul Ozyurt, Nese Merve Guner Zırıh.

Formal analysis: Aziz Gumus, Nur Hursoy.

Investigation: Aziz Gumus, Bilge Yılmaz Kara, Unal Sahin.

Methodology: Aziz Gumus, Neslihan Ozcelik, Songul Ozyurt, Nur Hursoy.

Supervision: Unal Sahin.

Writing-original draft: Aziz Gumus, Neslihan Ozcelik, Songul Ozyurt.

Writing-review & editing: Aziz Gumus, Neslihan Ozcelik, Songul Ozyurt, Bilge Yılmaz Kara.

All authors read and approved the final version of the manuscript.

### Funding

The authors declared that this case has received no financial support.

## Declaration of compting interest

The authors declare no conflicts of interest.
